# M. Biel on the Antiphlogistic Treatment of Cancer

**Published:** 1826-07

**Authors:** 


11. *Antiphlogistic Treatment of Cancer.* Mons. Puel, chief surgeon of the Civil Hospital of Figeac, had long been in the habit of treating, and, according to the documents published by his son, of *curing* cancer of the mamma and other parts, by starvation and bleeding, more especially local bleeding. The practice is not new, as every body knows—but still, it may be useful, though long neglected. When Dr. Barry shewed that cupping-glasses applied to a poisoned wound completely arrested the progress of the poison into the circulation, and secured the animal from certain death, it was immediately discovered that Celsus had recommended the same measure in poisoned wounds, and especially in those occasioned by the bite of a rabid animal. Yet, the recommendation of Celsus was a dead letter, to all intents and purposes, and, therefore, Dr. Barry is just as much entitled to the merit of the discovery as if Celsus had never put pen to paper. It is well known that Fearon, in this country, some 20 years ago, maintained that cancer was always an inflammatory affection, and that its only remedy was to be sought in repeated leechings, and the other antiphlogistic measures. Every well-informed surgeon, indeed, knows that the growth of cancerous, or rather of scirrhous tumours, is connected with, if not dependent on, an inflammatory or turgescent state of the vessels of the part, and, therefore, endeavours to check their growth by local depletion. But then, it must be recollected, that there is such a thing as a morbid *deposition* or change of structure, which is to be removed, and consequently absorption is to be promoted, while nutrition is to be retarded. This important process seems to have been over-looked both by Mr. Fearon, and M. Puel. In all tumours of the female mamma, and, indeed, we might say in all tumours, where there is pain, throbbing, or heat, leeches should be applied every three or four days, till the tenderness subsides, and then friction, with mild mercurial or iodine ointment should be alternated with the local depletion. This, with low diet and antimonials internally, will check or remove tumours if any thing will. Nine cases are related by the younger Puel, where the depletive practice was successful in the hands of his father, not only in scirrhus but in open cancer. These cases are detailed in the *Archives* for October, 1825.

